# Exclusion of Rasmussen Aneurysm: CT Technique

**DOI:** 10.5334/jbsr.4013

**Published:** 2025-08-18

**Authors:** Sander Gurdeep Singh, Piet Vanhoenacker

**Affiliations:** 1 UZ Gent, Corneel Heymanslaan 10, 9000 Gent, Belgium

**Keywords:** bronchial artery, embolization, recurrence haemoptysis

## Abstract

*Teaching point:* Pulmonary and systemic arterial phases are needed to exclude Rasmussen aneurysm.

## Case Report

A 36-year-old man presented to the emergency department with cough and episodes of haemoptysis. The patient had no fever or dyspnoea. Clinical history revealed tobacco use (20 pack-year) and recent unexplained weight loss. Clinical examination revealed crepitations in the right lung. The blood test showed an elevated CRP (53.4 mg/L) and normal white blood cell count.

Computed tomography (CT) was performed, and a thick-walled cavity was observed in the right upper lobe accompanied by alveolar consolidation and ground-glass opacities (GGO). A preliminary diagnosis of open tuberculosis (TBC) was suggested.

**Figure 1:**
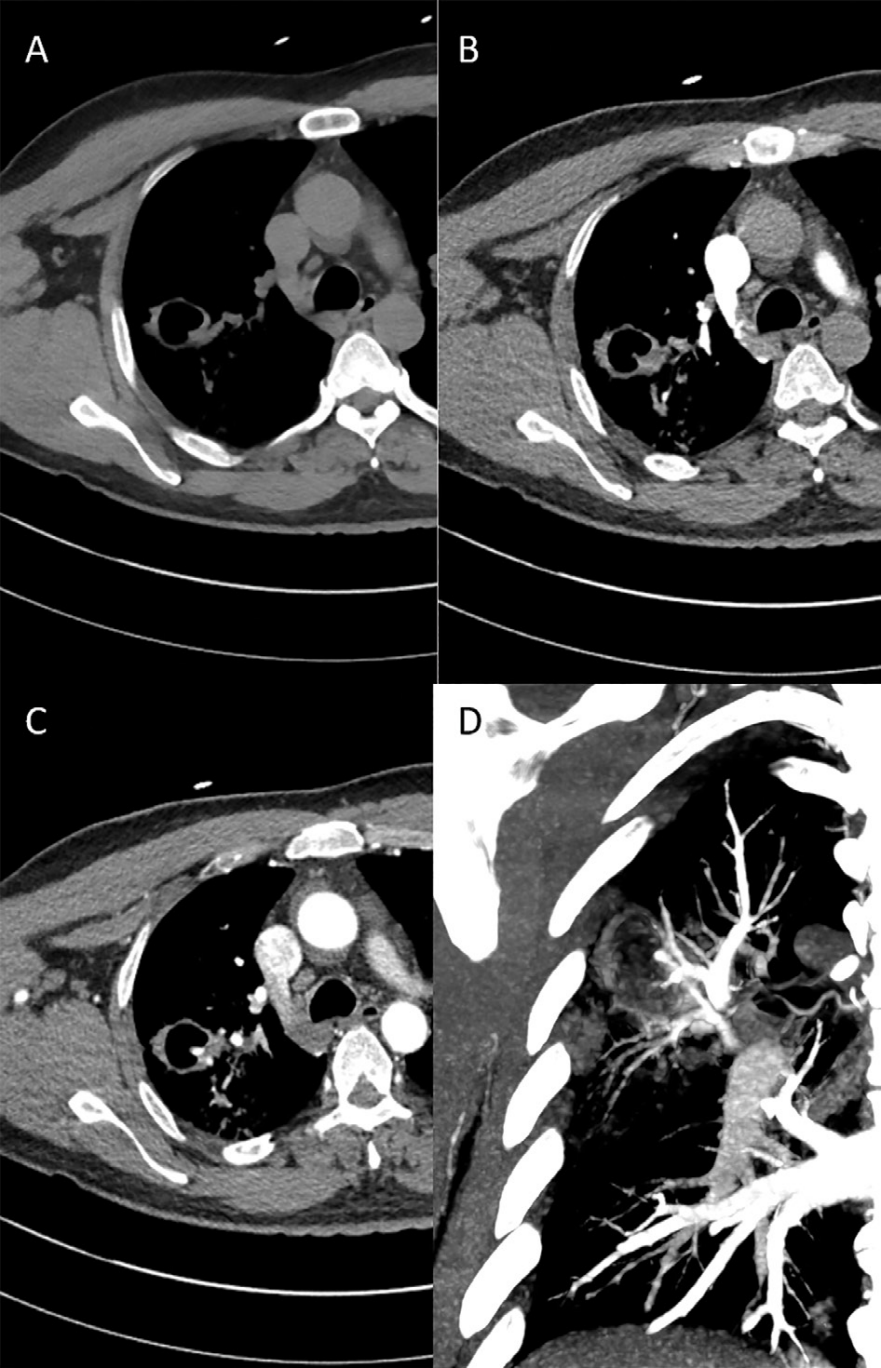
Non-enhanced CT **(A)**, CTA in pulmonary phase **(B)** and CTA in systemic arterial phase **(C)** of the cavernous lesion in the right upper lobe. **(D)** shows the 30 mm coronal MIP images of the systemic arterial phase **(C)**.

Thoracic CT angiography (CTA) of the lungs was performed in three phases: unenhanced (Figure 1A), contrast-enhanced in the pulmonary phase (Figure 1B) and in the systemic arterial phase (Figure 1C and D). A tick-walled cavity was seen in the right upper lobe with a nodular structure on the posteromedial side. The structure did not enhance in the pulmonary phase, but did enhance in the systemic arterial phase. The pulmonary veins surrounding the nodule show no enhancement in the pulmonary phase. A bronchial artery aneurysm was suggested.

**Figure 2:**
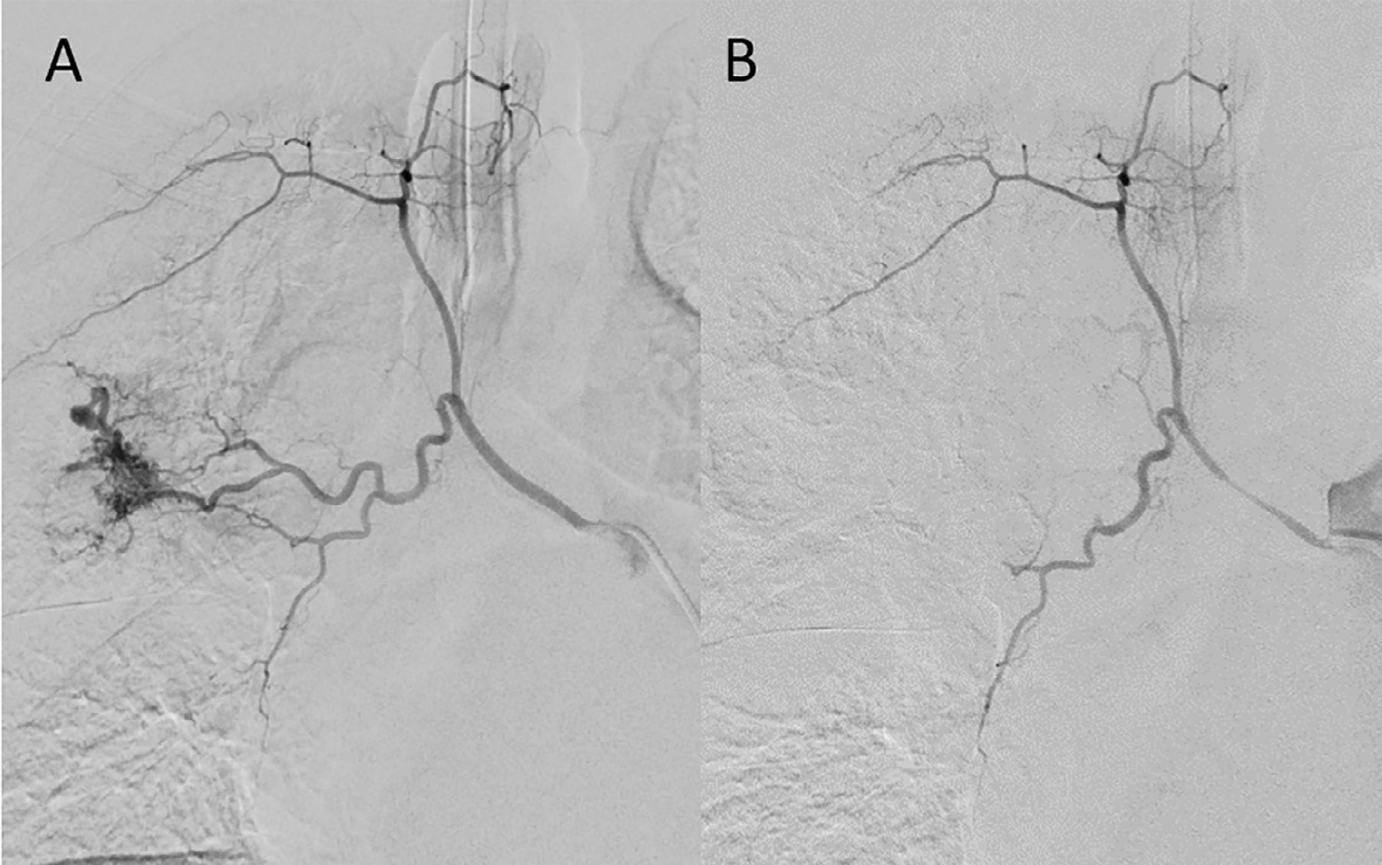
Digital subtraction angiography: Pre- and post-treatment selective angiography of the upper lobe branches of the pulmonary artery. **(A)** shows the pseudo-aneurysm in the right upper lobe. **(B)** shows the occlusion of the selective artery to the pseudo-aneurysm. Spasms of the bronchial artery are also depicted post-treatment.

A pseudo-aneurysm arising from the right bronchial artery was identified on a catheter angiography (Figure 2A). Treatment with glue-embolization was performed, after which no residual opacification was observed (Figure 2B).

## Comment

When evaluating a patient with haemoptysis, a possible but serious underlying cause is a vascular aneurysm, including a Rasmussen’s aneurysm from a pulmonary artery, and a systemic bronchial artery aneurysm. Correct identification of the aneurysm’s origin is critical, as it dictates both the angiographic approach and the interventional technique. With an appropriate CT-scanning procedure, the nature of aneurysm can be suggested. Such CT protocol consists of different phases [1]: (1) unenhanced phase as baseline to detect calcifications, high-density material (e.g., blood) and to differentiate vascular structures from clotted blood; (2) pulmonary arterial phase after i.v. contrast injection timed for the assessment of the pulmonary arteries; (3) systemic arterial phase for systemic arterial enhancement, especially bronchial arteries, intercostal arteries and other collaterals; (4) an optional systemic venous phase to evaluate venous drainage or delayed enhancement.

The final confirmative diagnosis was provided by catheter angiography. However, the specific CT protocol enabled the pre-procedural diagnosis. The diagnostic and therapeutic approach to angiography depends on the origin of the aneurysm. A pulmonary artery aneurysm (Rasmussen’s) requires a venous approach through the right heart with pulmonary artery cannulation, whereas a bronchial artery aneurysm needs an arterial approach.

## Conclusion

A structured CT protocol improves the diagnostic accuracy in cases of haemoptysis to differentiate between pulmonary and systemic sources. CT must aim to identify the aneurysm’s location and feeding vessel. Catheter angiography is both diagnostic and therapeutic. Multidisciplinary discussion, including the interventional technique, is preferable in planning therapeutic management.
